# The Use of Rubber-Polymer Composites in Bitumen Modification for the Disposal of Rubber and Polymer Waste

**DOI:** 10.3390/polym16223177

**Published:** 2024-11-14

**Authors:** Anar Akkenzheyeva, Viktors Haritonovs, Akkenzhe Bussurmanova, Remo Merijs-Meri, Yerzhan Imanbayev, Akmaral Serikbayeva, Serik Sydykov, Yerbolat Ayapbergenov, Martynas Jankauskas, Anatolijs Trumpels, Murshida Aimova, Maira Turkmenbayeva

**Affiliations:** 1 Engineering Faculty, Yessenov University, 32 Microdistrict, Aktau 130003, Kazakhstan; anar.akkenzheyeva@yu.edu.kz (A.A.); akmaral.serikbayeva@yu.edu.kz (A.S.); maira.turkmenbayeva@yu.edu (M.T.); 2 Faculty of Civil and Mechanical Engineering, Riga Technical University, 6A Kipsalas Street, LV-1048 Riga, Latvia; viktors.haritonovs@rtu.lv; 3 Science and Technology Faculty, Yessenov University, 32 Microdistrict, Aktau 130003, Kazakhstan; murshida.aimova@yu.edu.kz; 4 Faculty of Natural Sciences and Technologies, Riga Technical University, 3 Paula Valdena Street, LV-1048 Riga, Latvia; remo.merijs-meri@rtu.lv; 5 Laboratory of Petrochemical Processes, RSE “Institute of Combustion Problems”, Bogenbay Street, 172, Almaty 050012, Kazakhstan; erzhan.imanbayev@mail.ru; 6 LLP “JV” CASPI BITUM Aktau Bitumen Plant, Industrial Zone 5, Building 65, Aktau 130000, Kazakhstan; s.sydykov82@mail.ru; 7 Branch of LLP “KMG Engineering” “KazNIPImunaigas”, 35 Microdistrict, Section 6/1, Aktau 130000, Kazakhstan; ayapbergenov_e@kaznipi.kz; 8 JSC “Polylema”, Jegaines Str. 8, Kauno District, LT-54469 Biruliskes, Lithuania; info@polylema.lt; 9 LLC “Rubbintec”, Drosmes 2-34, LV-2015 Jurmala, Latvia; atrumpels@gmail.com

**Keywords:** petroleum bitumen, bitumen modification, crumb rubber, fluorescence microscopy, differential scanning calorimetry, thermogravimetric analyser

## Abstract

The use of rubber-polymer composites ELTC (End of Life Tire Compound) for bitumen modification was investigated. ELTC contains not only devulcanized rubber from used car tires, but also used plastics (polymers) such as polyethylene (PE) and polypropylene (PP). ELTC is obtained using the method of rubber devulcanization using a selective catalyst that allows selectively decomposing sulfide bonds at relatively low temperatures, while preserving most of the macromolecular chains. The characteristics of the asphalt binder improved after the modification of ELTC. After modification, the base asphalt binder became more homogeneous, and the thermal stability of the base asphalt binder increased. ELTC is evenly distributed, the compatibility between the components of the modified asphalt binder is good, which proves the uniformity of the modified asphalt binders. The results show that all ELTC formulations improve the softening temperature and increase their resistance to plastic deformation in the summer.

## 1. Introduction

Worldwide, sustainable development is an important need in the construction industry, and to this end, the use of waste in road construction is increasingly encouraged to reduce environmental impacts. A large number of original materials and technologies suitable for the construction and maintenance of road surfaces have been invented in the road infrastructure. Some of them are plastics and rubbers. Currently, the amount of various rubber waste and plastic waste is increasing. According to a study sanctioned by the World Business Council for Sustainable Development, in 2018, the United States and the EU produced about 3700 and 3400 kg of used tires, respectively [1]. It is predicted that by the end of 2030, the number of used tires worldwide will reach approximately 5 billion [2]. The current global annual accumulation of end-of-life plastic goods is approximately 150 million tons [3]. Their non-biodegradability and toxic components pose a serious threat to the environment [4,5,6]. The use of rubber and plastic waste for the production of modified asphalt plays an important role in resource conservation and environmental protection [7,8,9]. At the same time, the constant increase in the number of vehicles highlights the need for better quality roads and engineering design. Despite the many advantages offered by modern technologies for the production of modified binders, the materials obtained can increase the total cost of asphalt concrete mix by up to 40% [10,11].

Plastic and rubber waste can be used to partially replace conventional material to improve the desired mechanical characteristics for a particular road mix. Also, the active use of waste as bitumen modifiers is carried out in order to increase productivity, as well as reduce the cost of paving materials. Historically, crumb rubber has been used to produce modified asphalt for a very long time.

A large number of studies of rubber bitumen mixtures have shown that mixing rubber crumbs with petroleum asphalt can improve the characteristics of asphalt at high and low temperatures, viscoelastic characteristics and anti-aging properties, as well as rubber bitumen mixtures have a longer service life compared to conventional asphalt concrete mixtures, reduce traffic noise, slow down the appearance of reflective cracks, improve the adhesion of binders materials with fillers, improve the slip resistance of the road surface and wear resistance [12,13]. Many states around the world have already introduced the use of rubber crumbs for bitumen modification due to increased resistance to track and fatigue cracking of rubber-bitumen binders [14,15,16,17,18].

Nevertheless, the use of rubber-bitumen binders causes some problems, such as higher production and laying temperatures, additional energy consumption, increased greenhouse gas emissions, and the need for constant mixing to avoid phase separation [19,20]. To eliminate the above problems, various solutions have been investigated, such as the addition of additives and modifiers or the devulcanization of rubber [21]. Vulcanized rubber crumbs have attracted special attention because the structural transformation of rubber involved in the devulcanization process leads to the formation of a product that can simultaneously solve problems of both high viscosity and phase separation. However, the use of devulcanized rubber in asphalt binders has led to contradictory results in terms of mechanical properties and is therefore at the research stage [22,23,24,25].

On the other hand, with the development of the plastics industry, plastic-modified asphalt is already taking its place in the road materials market. Plastics can significantly improve the high-temperature characteristics of asphalt, but cannot improve the low-temperature characteristics of asphalt; instead plastics reduce the low-temperature characteristics of asphalt [26]. Polyethylene (PE) and polypropylene (PP) are the most attractive plastic wastes for the road industry and are among the most commonly used additives to reduce rut, and the effect is more obvious under heavy loads [27]. The authors [28] found that high-temperature characteristics, resistance to low-temperature cracking and resistance to deformation have significantly increased. Maharaj concluded that the particle size and polyethylene content are of great importance for resistance to fatigue cracking and rutting [29]. Modification with polyethylene reduced the residual deformation, and the modified asphalt mixture began to have excellent resistance to residual deformation [30,31]. The binder modified with PE and PP constantly demonstrates problems with phase stability due to reduced chemical compatibility between macromolecules and asphalt components [32,33,34]. Moreover, polyethylene particles will separate from the asphalt binder if stored improperly, which limits the use of polyethylene-modified asphalt binder [35]. In addition, there is no improvement in the low-temperature characteristics of the asphalt binder when mixed only with polyethylene particles [36].

In recent years, researchers have begun to study asphalt modified with rubber-plastic composites. The researchers found that some asphalt compositions modified with rubber-plastic composites had better characteristics at high temperatures than asphalt modified with SBS, but their addition reduced the low-temperature characteristics of asphalt [29,37,38]. Nizamuddin et al. studied how the use of innovative additives that improve compatibility can help stabilize recycled asphalt concrete mixtures of rubber and plastic during high-temperature storage [39]. Various scientists have studied the properties of bitumen binders modified with rubber-plastic composites [13,40,41,42] and some have found that asphalt modified with a rubber-plastic composite has no less high-temperature characteristics than asphalt modified with SBS, but also good low-temperature characteristics [43]. It was also found that when the ratio of rubber and plastic was 7:3, the powder of rubber waste and plastic waste had good compatibility; the modifier of the spent rubber-plastic composite has good compatibility with the main asphalt, significantly improving the road characteristics of the main asphalt [44]. Wang studied the combined modification of polyethylene and rubber. The result showed that after modification, the rheological properties of the asphalt binder improved in various temperature ranges. To obtain a stable modified asphalt binder, it is necessary to reduce the density difference and strengthen the interaction [45].

Some chemical agents used in the rubber devulcanization process, usually present in devulcanized rubber, may be useful to improve storage stability in the presence of plastics. Preliminary studies show that the use of devulcanized rubber in combination with plastic waste improves storage stability of bitumen modified by plastic waste, only due to the presence of plasticizers, compatibilizers and crosslinking agents [46].

Thus, it is necessary to further study the modification of bitumen with rubber-plastic composites or hybrid modifiers.

To assess the effect of the hybrid modifier on the modification of road bitumen, the results were compared with unmodified road bitumen, modified SBS road bitumen (the main competitor of rubber modifiers on the world market).

## 2. Materials and Methods

Modified bitumen B70/100 was used as the initial bitumen. The characteristics of petroleum bitumen B70/100 are shown in Table 1.

In this work, the End of Life Tire Compound (ELTC) material from “Polylema” JSC manufacturer is used. “Rubbintec” LLC has proposed a method for the devulcanization of rubber using a selective catalyst that allows the selective decomposition of sulfide bonds at relatively low temperatures, while preserving most of the macromolecular chains. The resulting crumb from devulcanized rubber is intended for use both in rubber mixtures in the manufacture of new rubber products and as a bitumen modifier. A group of companies (“Rubbintec” and “Polylema”) has developed and patented in the United States a unique tire recycling technology that allows you to completely extract rubber from tires and use it to produce almost any rubber products. A key part of the “Rubbintec” devulcanization process is the combination of a selective catalyst and a compatibilizer. The catalyst is designed to break sulfide bonds, and the compatibilizer helps it penetrate the rubber crumb. The second function of the catalyst is its assistance in “grafting” devulcanized rubber to other polymers and organic components.

“Rubbintec” technology allows starting the process of decomposition of rubber at a much lower temperature—thermomechanical cracking of rubber crumbs in a reactor with two screws. In addition, cracking at temperatures below 550 °C (preferably 350–500 °C) preserves a large number of the original double bonds present in diene rubbers.

**Figure 1:**
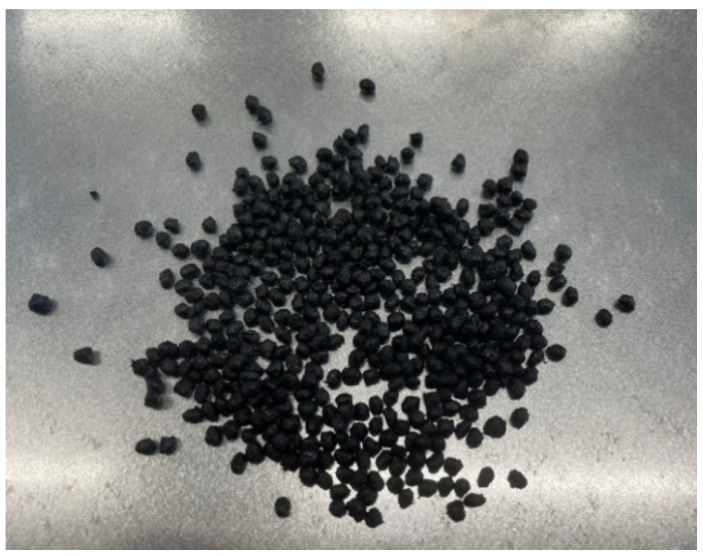
End of Life Tire Compound, developed by “Rubbintec” LLC and “Polylema” JSC.

ELTC technology is patented [53], and the copyright belongs to “Rubbintec” Ltd. The ELTC material is environmentally friendly. The strategy of the main enterprise of “Polylema” JSC is the development of a competitive material (increased flexibility at high and low operating temperatures will create a stable homogeneous bitumen and rubber system). Competitiveness must be achieved against alternatives available on the market, such as SBS (Styrene-butadiene-styrene) polymer, which is widely used to modify road bitumen. The cost of SBS polymer is about 3000 euros/ton, and ELTC is several times cheaper. The material produced by “Polylema” JSC is available in the form of granules with a diameter of up to 2.5 mm (see Figure 1).

The granules are black in color and consist of the following substances:

1. Crushed rubber from worn rubber tires 70–94%;
2. Thermoplastic polymer (waste), e.g., polypropylene or polyethylene 1–23%;
3. Additives 5–12%.

High Shear mixers are used in the laboratory to modify road bitumen. The production of modified road bitumen in the laboratory with the ELTC modifier of “Polylema” JSC consists of several stages:

1. Heating of road bitumen in an oven to 140 °C degrees;
2. The ELTC modifier is added (in parts) to 20% of the road bitumen mass and mixed at a speed of 6000 rpm and 170–180 °C degrees;
3. Mixing from 15 to 90 min;

A fluorescence microscopy (FM) Moticam PorS5Lite (OTICEUROPE, S.L.U., Barcelona, Spain ) was used to scan the target using fluorescence excitation filter D480/30 nm to observe distribution properties of targets according to the fluorescence shape.

Differential scanning calorimetry DSC-1 (Mettler Toledo, Greifensee, Switzerland) was used to record heating thermograms from −100 °C to 200 °C at scanning speed 10 °C in air environment. Average sample mass was 10 mg.

Thermogravimetric analyser TGA-1 (Mettler Toledo) was used to record decomposition thermograms from 20 °C to 800 °C at scanning speed 10 °C in air environment. Average sample mass was 10 mg.

## 3. Results and Discussion

Using the innovative ELTC modifier, several modified bitumen compositions were created in the laboratory. As ELTC is made of not only rubber from used car tires, but also used thermoplastics such as PE or PP, together with customized devulcanization additives it belongs to the group of hybrid modifier. In this modifier, rubber provides flexibility over the broad temperature range, while thermoplastics provide rigidity and act as glue (1) helping hold rubber particles in one monolith during the production process, (2) giving the product the shape of granules and (3) making it more suitable for further introduction in bitumen.

**Figure 2:**
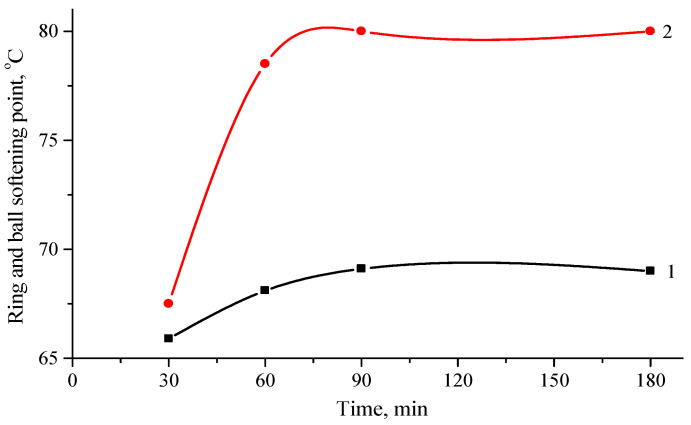
Change in softening temperature of ELTC + 10% PP modified bitumen from mixing time at different temperatures: 1—at 180 °C; 2—at 200 °C.

One of the most important functions of the modifier is to increase the softening temperature of bitumen, making the binder more resistant at high exploitation temperatures, which becomes important due to global temperature increase. Figure 2 shows the results for the softening temperature as a function of the temperature and time of high-shear mixing of the bitumen with 20 wt.% of ELTC + 10% PP modifier.

From the results shown in the Figure, it can be seen that all the investigated ELTC compositions improve the softening temperature. For the compositions obtained at 200 °C and mixing times over 60 min, softening temperatures are close to 80 °C, which means more than 20 °C increment compared to unmodified bitumen 70/100. Since the goal is to obtain modified bitumen with a softening temperature of at least 55 °C, the results show that the addition of the hybrid ELTC modifier not only allowed achieving this goal, but also exceeded it by a wide margin. It is important to note that in this study, the ELTC modifier was added to 20% of the bitumen mass. Also, in the previously reviewed literature [54,55], it was found that rubber improves the properties of bitumen by adding 15–25% by weight of bitumen. This denotes that large amount of rubber waste can be utilized in this way the cost of rubber-bitumen binders can be reduced. Also, the properties of modified bitumen were compared with the properties of bitumen modified with 4% SBS. The softening temperature with 4% modified SBS bitumen is 76.4 °C.

Bitumen modification was carried out at temperatures of 180 and 200 °C. These temperatures typical for bitumen modification at real production conditions. Figure 2 also shows that the modified bitumen was prepared in the laboratory using different mixing times within the range between 30 and 180 min. This was done in order to set a minimum modification time. It can be seen that the softening temperature rises to a stirring duration of 90 min, but then, when it reaches 180 min, there is no improvement. This means that it is enough to mix for 60–90 min. It is important to note that after 30 min, the softening temperature during mixing reaches 65.9 °C, which is a 17 °C degree higher than that of unmodified road bitumen B70/100.

**Figure 3:**
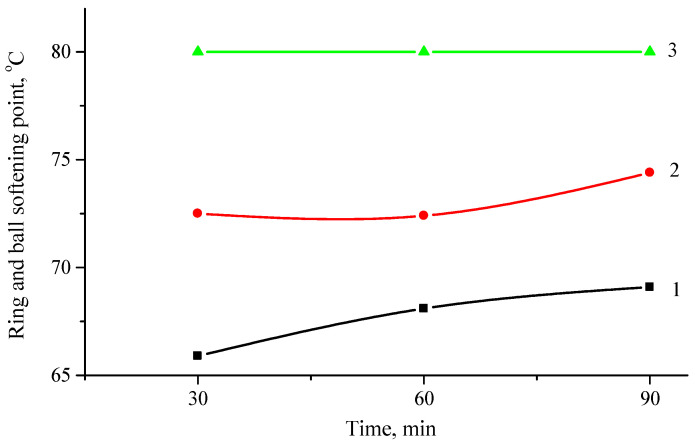
Change in softening temperature of ELTC modifier from mixing time at different concentration of PP: 1—at 10%; 2—at 20%; 3—at 30%.

The results show that a higher production temperature further improves the softening temperature, for example, when stirring at 180 °C for 90 min, the softening temperature is 69.1 °C and when stirring at 200 °C for 90 min–80 °C or almost 11 °C degrees higher. Figure 3 shows the effect of the PP content in the hybrid modifier on the softening temperature.

It is clearly seen that with an increase in PP from 10% to 20%, the softening temperature increases by 4.5–6 °C. On the other hand, with an increase in the polypropylene content by another 10 percent, i.e., up to 30%, the softening temperature increases by another 5.5–7 degrees, reaching 80 °C. As indicated in [56], the softening point can reveal the high-temperature stability of the asphalt binder. As we can see, when modifying bitumen with the hybrid ELTC modifier, the high-temperature characteristics of bitumen improve, whereas the improvement is larger by increasing the concentration of more rigid component in the modifier.

The authors of [57] also studied the effect of the concentration of used rubber tires and recycled polyethylene on the softening temperature of the modified asphalt binder. The softening temperature increased with an increase in the amount of rubber tires and polyethylene added to the base asphalt binder and improved performance at high temperatures. It is assumed that the mechanism of bitumen modification with the ELTC modifier is a combination of physical and chemical processes. As mentioned above, a key part of the “Rubbintec” devulcanization process is the use of a selective catalyst and a compatibilizer. This helps in the “grafting” of devulcanized rubber crumbs to the polymer and bitumen fractions, which helps to improve distribution of the ELTC modifier particles within asphalt binder matrix. In addition, as shown in [57], the TGA weight loss curves of a bitumen binder modified with used tire rubber and recycled PE remained largely unchanged, which proves that thermal stability improved after modification.

**Figure 4:**
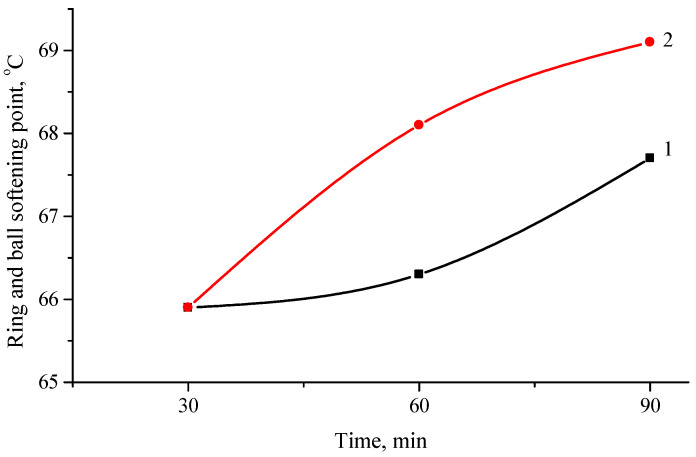
Dependence of softening temperature of ELTC modifier with 10 wt.% of PE (1) or PP (2) from mixing time.

PP and PE are the most common constituents or thermoplastic waste and both of these polymers usually are easily recyclable. However, due to their macromolecular structure both of these polymers do not have the necessary flexibility properties, especially at sub-zero temperatures, in contradiction to rubber or SBS polymers, which are widely used for bitumen modification. Therefore, in this study, PP and PE are used in combination with devulcanized rubber. The results show that there is not much difference between PE and PP as a constituent of ELTC modifier in terms of increment of softening temperature. However, somewhat higher increment of softening temperature is in the case of stiffer PP containing modifier. The softening temperature change is up to 1.8 °C (Figure 4).

**Figure 5:**
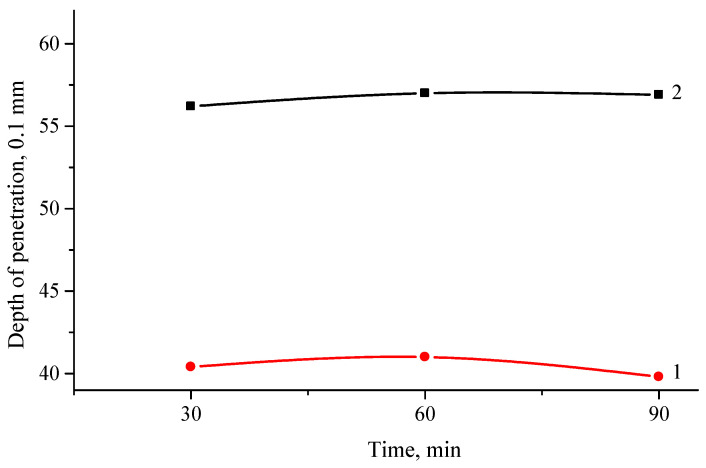
Change in penetration depth of ELTC modifier with 10 wt.% of PP (1) or PE (2) from mixing time.

Figure 5 shows the results of needle penetration at 25 °C of modified bitumen compositions, depending on the type of the polymer (PP and PE), present in the ELTC.

Needle penetration value for ELTC compositions containing 10% PP is about 40, and for PE—more than 56. Thus, the obtained results show that under the same production conditions, road bitumen containing ELTC with 10% PE is softer than ELTC with 10% PP, which is in accordance with stiffer macromolecular structure of the latter.

**Figure 6:**
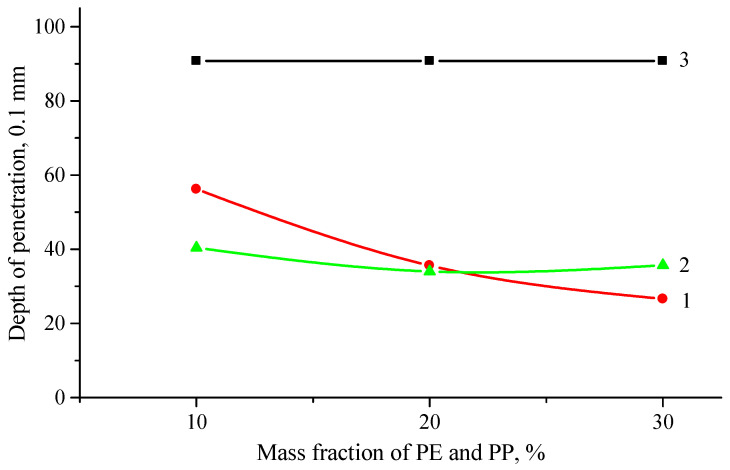
Change in penetration depth of ELTC modified bitumen binder (mixing time—30 min.; temperature—180 °C) with PE (1) and PP (2) in comparison to neat bitumen 70/100 (3) from the polymer concentration.

Figure 6 shows the effect of the thermoplastics content in the composition of ELTC on the results of needle penetration.

From the results obtained, it can be seen that with an increase in the polymer content, the penetrating ability of the needle decreases for all the investigated ELTC modified binders, denoting to increased stiffness of the system. This trend is especially pronounced for the bitumen with PE containing ELTC binder: at 10% PE content needle penetration value is 56.2, whereas at 40% PE content it is 26.6. On the other hand, in the case of PP, a considerable decrease in needle penetration was not observed.

The results show that the addition of modifiers reduces the penetration value from 90.8 to 26.6. This means that, the modified bitumen becomes harder compared to unmodified bitumen. The traditional class of modified bitumen B45/80-55, where 45/80 is minimum and maximum needle penetration values, can be achieved using the ELTC + 10% PE modifier, for these compositions the needle penetration values are within the range of 56.2–57. Most compositions have a score below 45, which means that they correspond to the bitumen class 25/55.

The penetration value of the asphalt binder may reflect the consistency of the shows the effect of used rubber tires and recycled PE on the penetration of modified asphalt binder. Penetration decreased after the addition of used rubber tires and recycled PE. It has been shown that some molecular composition of the asphalt binder have changed after the addition of used rubber tire crumb and recycled PE, and some physical reactions have occurred, as a result of which the asphalt binder has hardened and its penetration has decreased. In addition, as a result of the introduction of polymer waste, the viscosity of the system becomes greater, which reduces the depth of penetration of the needle.

**Figure 7:**
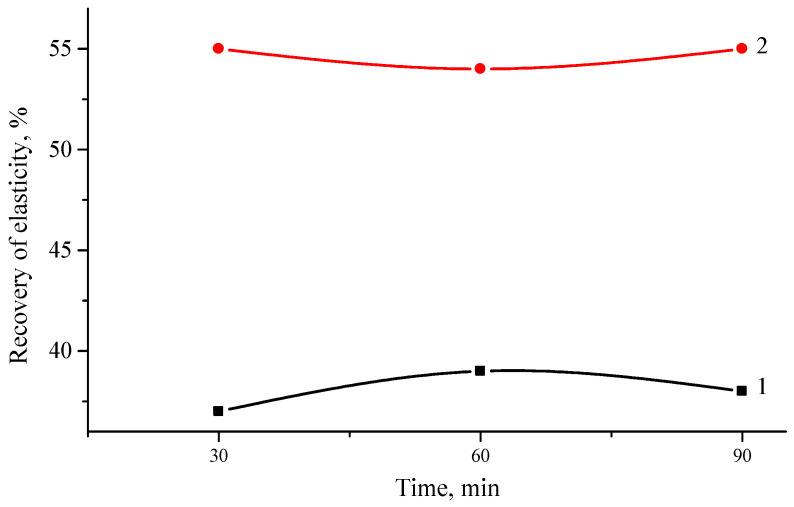
Change in of elastic recovery of ELTC modifier with 10 wt.% of PE (1) or PP (2) from mixing time.

Figure 7 shows the results of elastic recovery. This is a very important property of modified bitumen, as this parameter indicates whether the added modifier improves flexibility.

Previous results have shown that the modifier affects the properties of bitumen, making it tougher. On the other hand, hard material can cause brittleness or premature cracking. The results obtained show that hybrid ELTC modifiers containing PP have larger elastic recovery at 25 °C. It is important to note that unmodified bitumen 70/100 does not show elastic recovery under these test conditions, while the 4% SBS modified bitumen shows a 60% elastic recovery. The polymer-modified road bitumen should have an elastic recovery of 50% at least. This figure is achieved using the ELTC + 10% PP modifier—54–55%. Other compositions show significantly lower elastic recovery value, that is, about 30–40%. The paper [57] shows the effect of the concentration of used rubber tires crumb and recycled PE on the plasticity of the modified asphalt binder, while the plasticity decreased as the PE content increased, confirming that thermoplastics cannot improve the low-temperature characteristics of asphalt, instead the low-temperature characteristics of asphalt are reduced [26,36]. Plasticity increased with an increase in the content of rubber crumbs, this may be due to the fact that with a higher content of polymer waste and rubber crumbs, the interaction between them and the light component of the asphalt binder was stronger.

**Figure 8:**
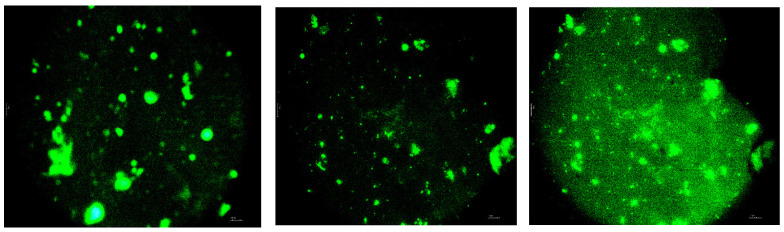
Morphology of modified bitumen binder with 20% of ELTC Mix 20/80PP mixed at 180 °C for 30 min (fracture surface of the sample, obtained by cooling the binder sample in liquid nitrogen).

**Figure 9:**
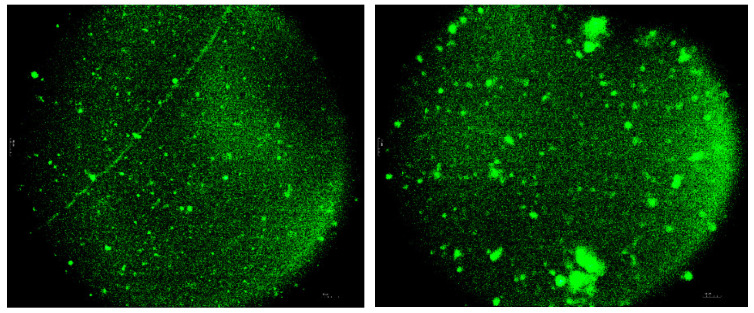
Morphology of modified bitumen binder with ELTC Mix 20/80PP 20% mixed at 180 °C for 90 min (fracture surface of the sample, obtained by cooling the binder sample in liquid nitrogen).

Figure 8 and Figure 9 show fluorescent micrographs of a modified asphalt binder, fluorescent micrographs can visually reflect the distribution of ELTC in asphalt binders.

Polymer-bitumen binder is considered as a material in which the medium is bitumen, and the dispersed phase is a polymer. It surpasses the properties of conventional bitumen. Microscopy studies of the investigated bitumen binder show that with certain amounts of polymer additive in bitumen binder, it is able to “dissolve”, but sometimes the polymer is distributed in bitumen in the form of separate, unregular particles. The effect of their action in the composition is similar to the effect of the filler. Mixing the composite for 30 min leads to distribution of ELTC binder in the bitumen in the form of separate particles. Mixing for 90 min leads to aggregation of particles and their fusion (Figure 8 and Figure 9), this leads to increased strength and elasticity of the resulting product.

According to the mechanism of ELTC modified bitumen, the ELTC particles are distributed in the asphalt matrix during high-shear mixing. The fluorescence occurs if the ELTC particles are lighted by electromagnetic irradiation at certain wavelength. Bitumen/asphalt phase is black and opaque and have no significant fluorescence reaction. The fluorescence reaction difference between the ELTC particles and bitumen matrix can be used to present the distribution properties. In FM images, the light-green part shows the ELTC and the black part shows neat bitumen/asphalt.

It can be observed:

(1) ELTC is dispersed in bitumen/asphalt in the form of roundish particles forming non-continuous phase.
(2) Some poorly dispersed agglomerated ELTC particles also are observed within bitumen/asphalt matrix.
(3) Increased mixing/dispergation time reduce the formation of agglomerates.
(4) The largest particle images observed during investigation are around 200 µm.
(5) Finely, dispersed ELTC particles distributed in bitumen homogeneously can have a positive effect on storage stability.
(6) Coarser undispersed particles are unevenly distributed in the system and influence storage stability.

The authors of the paper [57] noted that when mixed with recycled PE, the mutual adhesion of polyethylene and rubber crumbs improved the compatibility between polyethylene and the asphalt binder phase, since the rubber crumbs absorbed light molecular components from asphalt binders when they were mixed [58]. It was found that with the correct content of the modifying agent, rubber crumbs and recycled PE merge with asphalt binders and are evenly distributed, compatibility between the components of the modified asphalt binder is good, which proves the uniformity of the modified asphalt binders.

**Figure 10:**
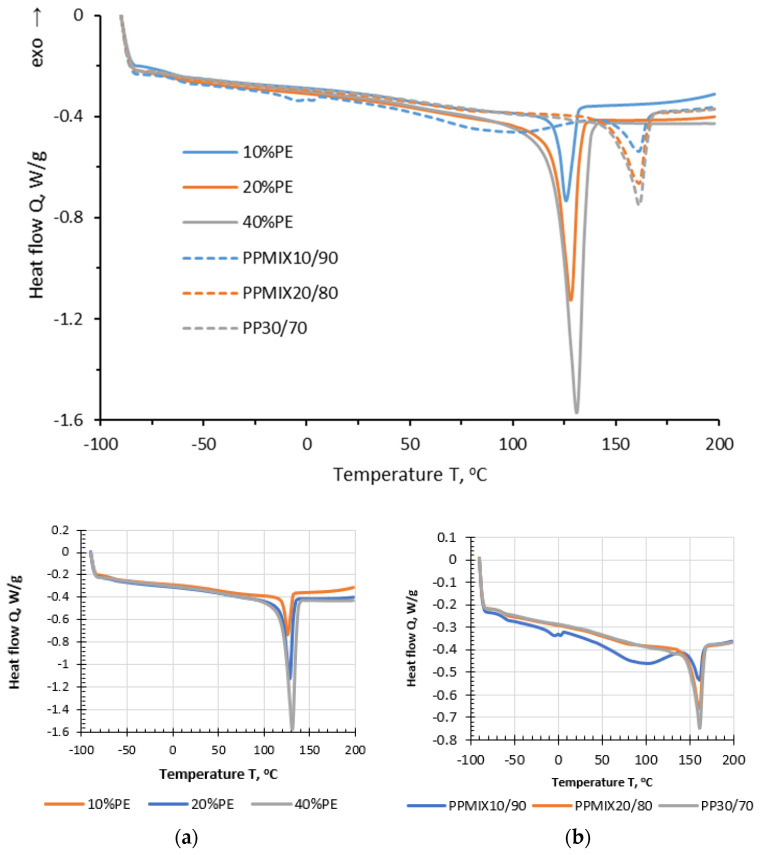
DSC thermograms of different ELTC modifiers based on PE (**a**) and PP (**b**).

By measuring the absorption and release of heat, DSC reveals the thermal characteristics of materials [34]. Table 2 demonstrates characteristic temperatures, whereas Figure 10 shows DSC thermograms of ELTC modifiers. 

As one can see melting peaks of PE based modifiers are considerably larger than in the case of PP based systems, which is explained by larger melting enthalpy of PE crystal (ca 290 J/g) in comparison of PP (ca 190 J/g). In the case of PE based modifier, widening of endothermic peak is observed by increasing the rubber content in the mixture denoting to restricted crystallization of the polyolefine phase. This complies well with decreased peak temperatures of PE crystalline phase by increasing the rubber content. In its turn, for PP based modifier, several endothermic effects are observed over the testing interval, especially for the composition with the highest rubber content. Consequently, the peaks centered around 0 °C and 93 °C are associated with rubber phase, denoting to presence of customized additives. It is evident that the endotherm with maxima around 93 °C, partly overlap with onset melting peak of PP crystalline phase. In spite of this overlap, maximum and offset melting peak temperatures of PP crystalline phase are not considerable affected by changing the rubber content. Most probably similar endothermic transitions of rubber phase are present also in the case of PE modifier; however, they are not observed due to peak overlapping and higher heat of fusion of PE crystal. Nevertheless, it is important to mention that melting of PE and PP crystalline fractions occurs below the used mixing temperature of ELTC with 70/100 grade bitumen.

**Figure 11:**
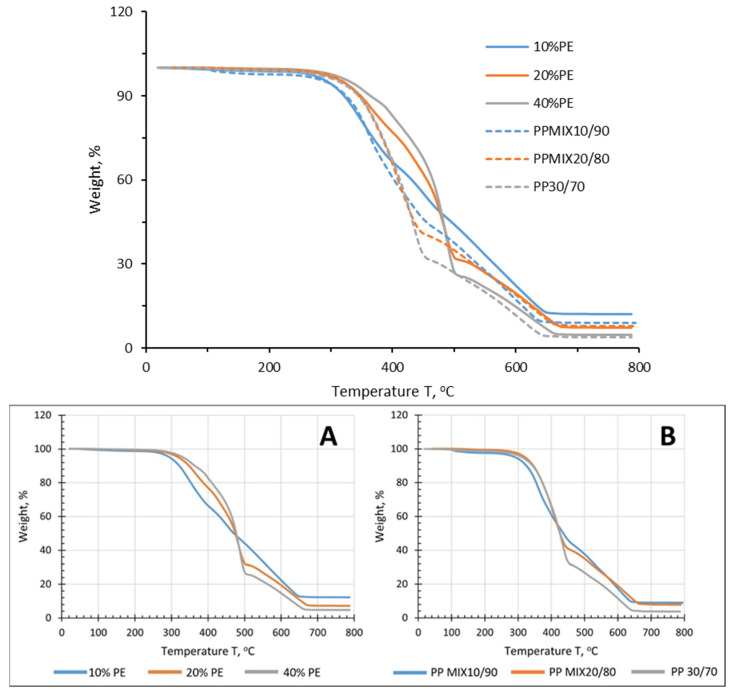
TGA thermograms of different ELTC modifiers based on PE (**A**) and PP (**B**).

TGA is a good indicator for assessing the thermal stability of an ELTC modifier [35]. Figure 11 depicts TGA thermograms, recorded by performing measurements under air atmosphere. Sufficient thermal stability of the investigated ELTC modifiers for compounding with bitumen or any other suitable constituent (e.g., thermoplastic) is observed by considering, if sufficient compatibility is ensured, i.e., main thermal destruction occurs above 250 °C.

According to the TGA graphs, ELTC modifiers decompose during several stages. Minor mass loss until 250 °C is associated with elimination of most volatile compounds. Considerable destruction of the binders starts only if heated above 300 °C. Comparatively thermal stability of PP based ELTC modifier is lower in comparison to than of PE based counterpart. This is evidently associated with the presence of tertiary carbon atoms in the PP macromolecule, which is more susceptible to thermal degradation. By increasing polyolefine concentration up to 40% onset of TGA curves is shifted towards direction of higher temperatures. Concomitant the first mass loss stage is shifted towards higher temperatures by increasing the polyolefine content. Increment in the first mass loss stage also may be observed by increasing the polyolefine concentration, which testify, that this mass loss stage is mainly associated with decomposition of PP or PE in the ELTC modifier. Consequently, decomposition rate of ELTC modifier becomes slower if rubber content is increased, resulting in larger char yields at the end of the experiment, especially in the case of PE-ELTC.

## 4. Conclusions

Experimental results show that all ELTC compositions improve the softening temperature and increase their resistance to plastic deformation in the summer. Some compositions, such as ELTC + 20% PE and ELTC + 30% PE have a softening temperature of 80 °C, which means an increase in the softening temperature by more than 20° C compared to conventional unmodified bitumen B 70/100. The highest improvement is explained by more rigid macromolecular structure of PE and especially PP in comparison to partially devulcanized rubber phase.

The results of the needle penetration test at 25° C show that the addition of ELTC modifiers reduces the penetration value from 90.8 to 26.6 at the maximum. This means that, the modified bitumen becomes harder compared to unmodified bitumen, which is explained predominantly because of chain rigidity of the polyolefine phase.

The criteria of polymer modified road bitumen in respects of elastic recovery of 50% in accordance with EN 14023 can be achieved by adding 20% of the ELTC modifier with 10%PP to B 70/100 road bitumen, demonstrating elastic recovery value of 54–55%. Other compositions show significantly lower elasticity recovery values, i.e., about 30–40 percent.

Important, that by increasing mixing/dispergation time of bitumen with ELTC modifiers it is possible to reduce the formation of agglomerates, enabling to obtain more regular distribution of the binder within bituminous matrix.

It is also important to mention that mixing of bitumen with ELTC modifiers occurred above melting of crystalline phases of both thermoplastic polymers (PE and PP). Concomitant, no considerable thermal destruction of the ELTC modifiers occurs at the mixing temperatures (180 °C and 200 °C) used for preparation of the modified bitumen binder.

## Figures and Tables

**Table 1:** Characteristics of petroleum road viscous bitumen grade B 70/100.

Bitumen Properties	Normative Indicators of the Road Bitumen B 70/100	Actual Value	Test Method
Penetration at 25 °C, not lower	87 ± 5	87.2	EN 1426:2015 [47]
Softening point °C, not below	45.8 ± 1.6	45.85	EN 1427:2015 [48]
Brittleness temperature on Fraas °C, not higher	–21 ± 3	–21	EN 12593:2015 [49]
Solubility %, not less	99.75 ± 0.1	99.75	EN 12592:2015 [50]
Flash point °C, not below	334 ± 4	335	EN 2592:2006 [51]
Rolling Thin Film Oven Test (RTFOT)			
Change of Mass	−0.021 ± 0.01	−0.0212	EN 12607-1:2015 [52]
Increasing in Softening Point	7.0 ± 3.6	7.0	EN 1427:2015 [48]
Decreasing in Softening Point	52.8 ± 1.6	53.25	EN 1427:2015 [48]
Retained Penetration	52 ± 3	51.9	EN 1426:2015 [47]

**Table 2:** Main characteristics of the polyolefine phase of the ELTC modifiers.

ELTC Modifier	Onset Melting Temperature, °C	Peak Melting Temperature, °C	Offset Melting Temperature, °C	Peak Width, °C
10%PE	67	126	136	69
20%PE	85	128	143	58
40%PE	94	131	146	52
PPMIX10/90	Peak overlapping	160	172	Peak overlapping
PPMIX20/80	Peak overlapping	161	174	Peak overlapping
PP30/70	Peak overlapping	161	173	Peak overlapping

## Data Availability

Data are contained within the article.
